# Multifunctional Nanographene Oxide for Targeted Gene-Mediated Thermochemotherapy of Drug-resistant Tumour

**DOI:** 10.1038/srep43506

**Published:** 2017-03-08

**Authors:** Yiping Zeng, Zhangyou Yang, Hong Li, Yuhui Hao, Cong Liu, Lin Zhu, Jing Liu, Binghui Lu, Rong Li

**Affiliations:** 1 State Key Laboratory of Trauma Burns and Combined Injury, Institute of Combined Injury, Chongqing Engineering Research Center for Nanomedicine, College of Preventive Medicine, Third Military Medical University, Chongqing 400038, China

## Abstract

Drug resistance remains a major challenge for anticancer treatment, and one of the major mechanisms of drug resistance is the overexpression of drug efflux transporters in cancer. A new approach for defeating drug resistance is the use of a co-delivery strategy that utilizes small interfering RNA (siRNA) to silence the expression of efflux transporters together with a suitable anticancer drug for drug-resistant cells. In this work, multifunctional graphene capable of integrating multiple functions in one system was employed as a novel co-delivery system for siRNA and doxorubicin (Dox), as well as for the controlled release of intracellular pH-triggered and heat-triggered Dox. Additionally, it was used as a synergistic therapy based on the photothermal effect of graphene oxide (GO) under near-infrared (NIR) irradiation and the chemotherapeutic effect of Dox. The nanocomplex exhibited high drug and siRNA loading. Furthermore, the dual delivery of siRNA and Dox by folic acid (FA)-conjugated polyethylenimine-modified PEGylated nanographene (PPG-FA/siRNA/Dox) exhibited a satisfactory gene silencing effect as well as efficient intracellular delivery of Dox. Thus, Dox could access the nucleus and induce greater cytotoxicity compared with siRNA-absent delivery systems. Significantly, under irradiation, the combined treatment showed more synergistic effect for overcoming drug resistance compared with chemotherapy effect alone.

Currently, cancer is one of the major causes of mortality and morbidity in the world, and chemotherapy remains is the primary conventional therapy for cancer. However, the anticancer efficacy of chemotherapy drugs can be severely limited by the development of multiple drug resistance (MDR) in cancer treatment^1^,^2^,^3^,^4^. Cancer cells exhibit MDR to many conventional chemotherapy drugs, and this behaviour is commonly associated with the overexpression of drug efflux transporters, such as P-glycoprotein (P-gp), which is a cell membrane protein involved in MDR. P-gp is constitutively expressed in normal cells, selectively overexpressed in carcinomas, and a member of the ATP-binding cassette (ABC) transporter family, and it can increase the efflux of various hydrophobic anticancer drugs^5^,^6^,^7^. Although considerable efforts have been devoted to developing a combination of chemotherapeutic agents to prevent drug resistance in cancer patients, however, they didn’t succeed in reversing MDR in clinical due to certain unwanted side effects and unpredictable pharmacokinetic interactions with anticancer drugs^8^,^9^.

The small interfering RNA (siRNA) technique has the potential to be developed into a more powerful method of reversing MDR in cancer cells; because it can disrupt cellular MDR pathways by silencing relevant gene expression, which can re-sensitize cancer cells that have acquired resistance to anticancer drugs^10^,^11^,^12^. However, it faces many obstacles for the application of siRNA, such as elimination, ribonuclease degradation, poor permeability and endosomal trapping. Therefore, the delivery of siRNA to tumourigenic P-gp upon systemic administration remains a major challenge^13^. The delivery system for tumour-targeted siRNA delivery upon systemic administration should be designed to overcome known hurdles, such as poor stability, low cellular uptake, and rapid siRNA clearance from circulation. It is shown that nanocarriers have the ability to protect the siRNA and mediate efficient gene silencing in cancer cells. An additional advantage of nanocarriers is their ability to deliver chemotherapy drugs^14^. Synergistic gene and drug therapy in the same nanocarrier is a promising strategy for treating cancer cells exhibiting MDR. Smart nanocarriers that are responsive to external stimuli, such as heat, glutathione, light, pH or magnetic fields, are also necessary for the controlled release of genes or drugs at suitable sites^15^,^16^,^17^,^18^.

Several types of nanocarriers, containing carbon nanotubes, liposomes, graphene oxide (GO), silicon nanomaterial and polymer micelles have been developed to co-deliver siRNA and chemotherapy drugs both *in vitro* and *in vivo* to enhance therapeutic action against cancer cells exhibiting MDR in recent years^19^,^20^,^21^,^22^. Among the nanocarriers, graphene oxide nanocarrier could be used for chemotherapy drug delivery, because of its good biocompatibility, high drug-loading capacity, and adsorption of aromatic drug molecules through π–π stacking and hydrophobic interaction, as well as the surface can be functionalized easily^23^,^24^,^25^,^26^. Polyethyleneimine (PEI), which is a cationic polymer, was used for the attachment of a series of siRNAs *via* electrostatic adsorption. Based on our previous research^27^,^28^, the carboxylic acid functional groups located at the edge of polyethylene glycol (PEG)-modified nanographene (NGO-PEG:PG) should react with PEI *via* covalent interaction. Thus, the cationic surface of the polyethyleneimine-coated graphene (PPG) could be used to co-deliver the chemotherapy drug and siRNA. More importantly, by utilizing their intrinsic high near-infrared (NIR) absorbance, GO and functionalized NGO have also been used as photothermal agents for *in vivo* cancer treatment with encouraging therapeutic outcomes^29^,^30^.

The goal of the present study was to develop an integrated NGO nanodelivery system for the efficient and targeted co-delivery of doxorubicin (Dox) and siRNA against P-gp expression to overcome drug-resistant tumour under 808 nm irradiation. The MCF-7/ADR cell line was used as a drug-resistant cell model that overexpresses P-gp, which represents one of the major mechanisms of MDR in cancer. Hence, based on the results of our previous study^27^,^28^, low molecular weight branched polyethylenimine (1800) modified PEGylated nanographene (PPG_1800_) was prepared successfully for photodynamic therapy (PDT). In this study, we used high molecular weight (10000) instead of low molecular weight branched polyethylenimine to modify the PG and yield more positively charged particles that strongly bind to negatively charged siRNA *via* electrostatic interactions. More importantly, the amine group of PPG could react with the carboxylic acid of folic acid (FA) *via* covalent interactions. Hence, the FA-conjugated high molecular weight branched polyethylenimine (10000)-modified PEGylated nanographene (PPG-FA) was prepared as a targeted co-delivery system for siRNA and Dox. In addition, the PPG-FA/siRNA/Dox system is a thermal-responsive co-delivery system because of the intrinsic high NIR absorbance of NGO. The schematic illustration of the PPG-FA nanocarrier for the intercellular co-delivery of siRNA and Dox into MDR cancer cells under 808 nm irradiation is shown in Fig. 1. We demonstrated that the siRNA co-delivery system produced a dramatic reversal of the resistance to Dox, increased intracellular Dox concentration with improved cytotoxicity and allowed the nuclear accumulation of released Dox in the drug-resistant cells. The drug-release kinetics, cellular uptake, *in vitro* cytotoxicity with or without irradiation, and intracellular localization were evaluated to demonstrate that the thermal-responsive PPG-FA/siRNA/Dox system is an excellent nanocomplex for the effective killing of drug-resistant cells.

## Results

### Preparation and characterization of PPG-FA/PPG-FA-Dox

**Figure 1:**
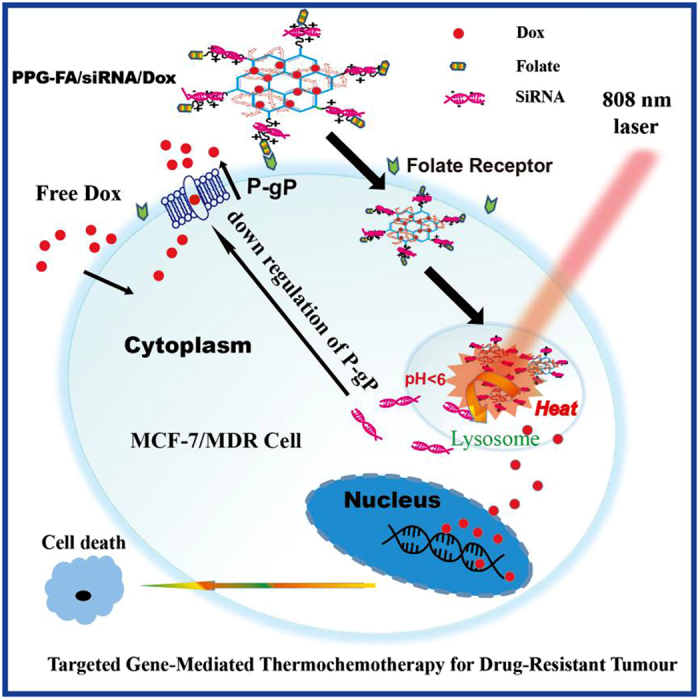
Targeted gene-mediated thermochemotherapy for drug-resistant tumour.

**Figure 2:**
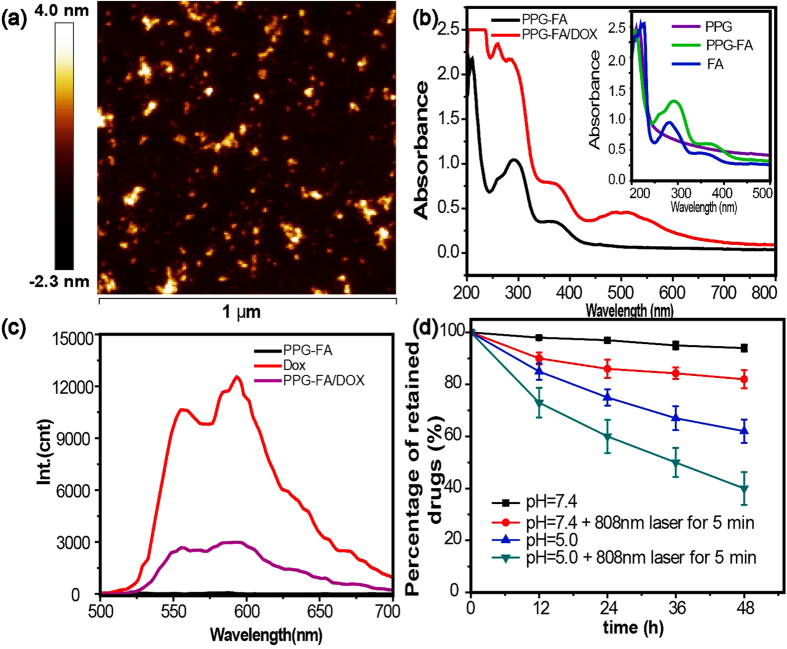
AFM image of PPG-FA/Dox (**a**), (**b**) UV/vis absorbance spectra of PPG-FA and PPG-FA-Dox, inset: FA, PPG and PPG-FA. (**c**) Fluorescence spectra of PPG-FA/Dox, Dox, and PPG-FA. (**d**) Dox release from PPG-FA/Dox at different pH values (7.4 and 5.0) with or without 808nm laser (2 W for 5min). Data are presented as the mean values ± S.D (n = 3).

PPG was prepared from PG as described in our previous report^27^. The desired carrier PPG-FA conjugate was formed *via* amide bond formation between the amino groups of PPG and the carboxyl groups of FA by N-(3-dimethylaminopropyl)-N -ethylcarbodiimide (EDC)/N-hydroxysuccinimide (NHS) coupling. The inset of Fig. 1b shows the absorption spectra of FA, PPG, and PPG-FA in water, and the successful conjugation is clearly evident from the peak at 283 nm in the UV/vis spectral analysis. Dox was also successfully loaded on the plane of PPG-FA *via* π–π stacking and hydrophobic interactions, and the absorption peak of Dox at 490 nm is shown in the UV/vis spectral analysis (Fig. 2b). The structural characterization and morphology of PPG-FA/Dox were characterized by atomic force microscopy (AFM) and transmission electron microscopy (TEM). As shown in the AFM and TEM images (Fig. 2a and Fig. S1), the size and thickness of the PPG-FA/Dox were 20–40 nm and ~ 2 nm respectively. These results indicated that FA and Dox were successfully conjugated on the PPG.

The fluorescence spectra of PPG-FA-Dox, PPG-FA and free Dox at the same Dox concentration were recorded *via* red fluorescence. As shown in Fig. 2c, the emission wavelength of Dox was 520–640 nm, however, PPG-FA emissions were not observed at the same position. However, the fluorescence of PPG-FA/Dox was quenched once Dox attached onto PPG-FA, thus revealing the excited-state interactions of Dox and PPG-FA driven by hydrophobic interactions and π-π stacking.

The photothermal properties of graphene and GO have been previously reported^29^,^30^. The heat generated by PPG-FA upon NIR irradiation was evaluated to confirm the thermal energy conversion from NIR light energy. As shown in Fig. S2, the temperature increased over time and reached approximately 43 °C after 10 min at NGO concentration of 15 μg/mL. Because of the high NIR absorbance contributed by GO, the PPG-FA exhibited a rapid temperature rise under irradiation by an 808 nm laser, suggesting the considerable photothermal capacity of PPG-FA.

Dox is known to have pH-dependent hydrophilicity^31^. At acidic pH conditions, Dox became more water soluble due to the protonated daunosamine group, which does not facilitate the hydrophobic π–π stacking interaction with PPG-FA. In contrast, under basic condition, the deprotonated Dox is hydrophobic, a characteristic that is beneficial for its effective π–π stacking interaction with PPG-FA^32^. Due to the pH-dependent π–π stacking interaction between Dox and PPG-FA, the drug release at different pH conditions was investigated at pH 5.0 and 7.4, which represent the acidic tumour microenvironment and physiological environment, respectively. Furthermore, the drug release mediated by the photothermal effect under acidic condition (pH = 5.0) and neutral condition (pH = 7.4) was also studied. As shown in Fig. 2d, it displayed low natural drug release behavior at neutral pH (pH = 7.4) without irradiation treatment. Only 10% of the Dox was released after 48 h. However, while applying 808 nm laser, which lead to the more release of Dox from the PPG-FA. The results indicated that the release rate can be obviously increased while applying NIR irradiation. The release profile in acidic condition (pH = 5.0) has a similar trend, while the released amount of Dox is higher than that in neutral pH with or without irradiation. Actually, the drug release at pH 5.0 reached to 60% at 48 h after laser irradiation, only 40% was released at pH 5.0 without irradiation. The results exhibited that both pH condition and photothermal effect could facilitate the drug release from the graphene nanocarrier. The pH-sensitive release demonstrated by the carrier could be ideal for anticancer therapy because both the extracellular microenvironments of tumours tissues, intracellular lysosomes and endosomes are acidic.

### Intracellular localization

**Figure 3:**
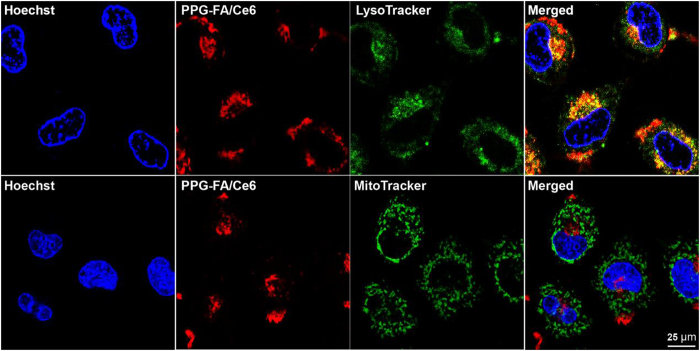
Localization of PPG-FA/Ce6 with a lysosome tracker (LysoTracker) or a mitochondria tracker (MitoTracker) in the MCF-7/ADR cells as imaged via confocal microscopy.

Confocal laser scanning microscope (CLSM) was used to provide visual evidence of the intracellular localization of PPG-FA. Because the Dox released from PPG-FA/Dox is capable of reaching and may interfere with the localization experiment; therefore, we uses chlorin e6 (Ce6) instead of Dox to react with the PPF-FA and the localization was observed utilizing the fluorescence of Ce6. MCF-7/ADR cells were incubated with PPG-FA/Ce6 and stained with lysosome tracker (LysoTracker), mitochondria tracker (MitoTracker) and cell nucleus tracker (Hoechst). As shown in Fig. 3, the CLSM results did not reveal an overlap of yellow/orange between the MitoTracker (green) and the fluorescence signals from the PPG-FA/Ce6 (red), suggesting that the PPG-FA/Ce6 was not localized in the mitochondria. Interestingly, the fluorescence signal from the LysoTracker (green) was consistent with the fluorescence signal from the PPG-FA/Ce6 (red), suggesting that most of the PPG-FA/Ce6 was localized in the lysosomes indicated by the yellow/orange overlap yield. Moreover, the acidic microenvironment of the lysosome is beneficial to the release of the drug from the carrier to enhance the chemotherapeutic efficacy. More importantly, lysosomes have been shown to be labile organelles that are highly sensible for photothermal therapy^33^,^34^.

### Internalization of PPG-FA/siRNA-FAM and *in vitro* gene silencing efficiency

**Figure 4:**
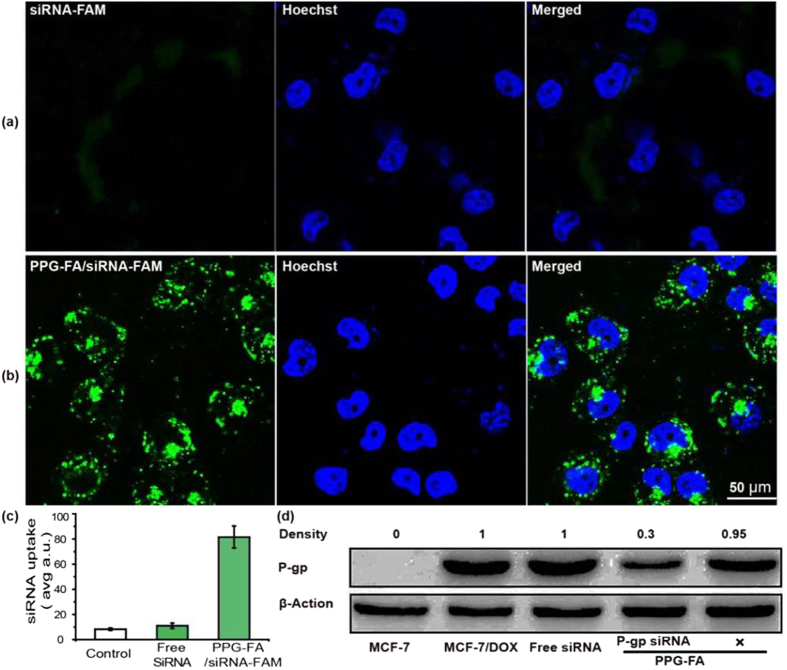
CLSM images of the MCF-7/ADR cells incubated with siRNA-FAM (**a**) and PPG-FA/siRNA-FAM (**b**). Cellular uptake amount of siRNA in the MCF-7/ADR cells after incubating for 4 h (**c**). Detection of P-gp knockdown by PPG-FA/siRNA using Western blotting (**d**). The relevant P-gp expression was calculated by the signal intensity of the protein bands. “X” denotes for cells treated by scrambled PPG-FA/siRNA.

In this study, PPG-FA was designed to co-delivery the anticancer drug Dox and siRNA into drug-resistance cells. Based on the above mentioned results, Dox was successfully loaded on the PPG-FA *via* π–π stacking interaction. In general, it is known that primary amino groups could condense siRNA better than the other forms of amines^35^. Additionally, the sufficient protection of the cargo from nuclease degradation was required for the successful siRNA delivery. Because of the cytotoxicity of PPG-FA/Dox, it was replaced by PPG-FA (drug free) to complex with siRNA, which was validated by a cellular uptake analysis and Western blot assay. The PPG-FA not only efficiently protected siRNA from nuclease degradation, but also provided abundant primary amino groups to bind siRNA. To investigate the cellular uptake of PPG-FA/siRNA, FAM-labelled siRNA (siRNA-FAM) was reacted with the PPG-FA. The nuclei of the cells were stained with Hoechst, and the siRNA-FAM was regarded as the control group. Green fluorescence observed in the cytoplasm indicated intracellular uptake of the PPG-FA/siRNA-FAM or siRNA-FAM. As shown in Fig. 4a and b, the CLSM images revealed that the PPG-FA/siRNA-FAM was mostly localized in the cytoplasm, and siRNA-FAM rarely entered the MCF-7/ADR cell, thus demonstrating that siRNA was stably attached to the PPG-FA particle surface. A significant increase in siRNA uptake was confirmed by an Image J software analysis after transfection with the PPG-FA/siRNA-FAM compared with that following transfection with the siRNA-FAM (Fig. 4c). This high uptake was caused by the positively charged amine group of PEI binds siRNA stably, and is resistant to enzymatic cleavage. The efficient knockdown of P-gp expression usually increases the sensitivity of tumour cells to anticancer drugs. Therefore, to evaluate the efficient delivery of P-gp siRNA delivered by PPG-FA, P-gp knockdown was confirmed by Western blotting. PPG-FA/scrambled siRNA (labelled as “X” in Fig. 4d) was served as a negative control. The results indicated that P-gp knockdown did not occur in the PPG-FA/scrambled siRNA group, or in the negative siRNA group. However, a 70% decrease in protein expression was observed in the PPG-FA/siRNA group compared with that in the control groups, suggesting that PPG-FA/siRNA can effectively knockdown the P-gp expression level to enhance the entrance ability of anticancer drugs.

### Cellular uptake of PPG-FA/siRNA/Dox

**Figure 5:**
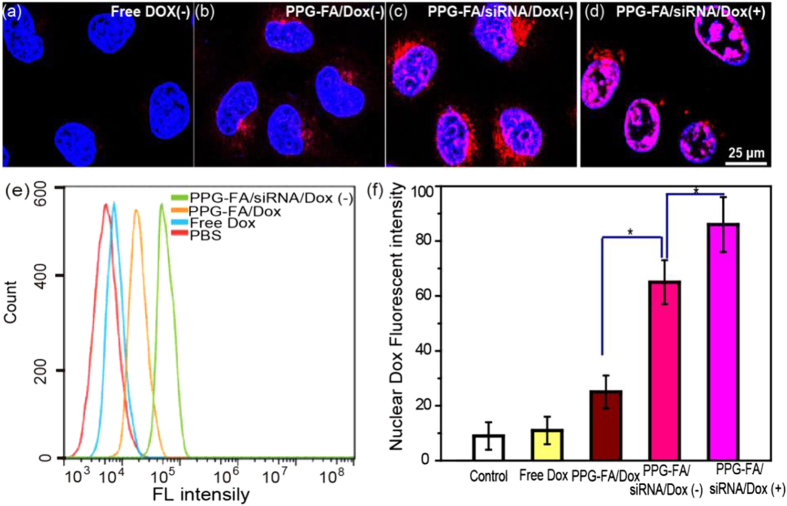
CLSM images of MCF-7/ADR cells treated with Dox (**a**), PPG-FA/Dox (**b**), PPG-FA/siRNA/Dox (**c**) and PPG-FA/siRNA/Dox with irradiation (**d**). Cell uptake of PBS, Dox, PPG-FA/Dox and PPG-FA/siRNA/Dox for 24 h was measured by flow cytometry (**e**). The fluorescence signal in MCF-7/ADR nuclei was quantificationally analyzed by Image J software (**f**). P values in (**f**) were calculated by Tukey’s post-test (*P < 0.05).

Compared with the siRNA uptake in the free siRNA solution, the uptake of PPG-FA/siRNA was significantly enhanced, indicating that the siRNA internalization could be assisted by PPG-FA/siRNA. We believe that the PPG-FA/siRNA also could promote the Dox internalization which was corroborated by the confocal imaging. As shown in Fig. 5, significant red fluorescence was not observed when the cells were treated with free Dox (Fig. 5a). Compared with free Dox, the low drug uptake ability was slightly improved after treatment with PPG-FA/Dox (Fig. 5b). It is interesting that the intracellular Dox fluorescence intensity was increased dramatically in the existence of siRNA (Fig. 5c). In addition, the strongest red fluorescence was observed when the cells were treated with PPG-FA/siRNA/Dox after 808 laser irradiation at 20 h (Fig. 5d); the reason may be that the photothermal effect induced more Dox release from PPF-FA/siRNA. The flow cytometry was used to determine intracellular Dox concentration *via* measuring the intracellular fluorescence intensity of Dox (Fig. 5e). The MCF-7/ADR cells treated with PPG-FA/siRNA/Dox showed a significant increase in the fluorescence signal compared with those treated with phosphate-buffered saline (PBS), free Dox and PPG-FA/Dox; however, a maximum 2-fold increase was achieved with the PPG-FA/siRNA/Dox treatment relative to the PPG-FA/Dox treatment. This finding suggests that knocking down P-gp expression increase the drug concentration in the MCF-7/ADR cells, which is consistent with the phenomenon obtained by CLSM. The acidic pH condition of lysosomes allowed for the release of Dox from the carriers and its entry into the nucleus. Hence, Image J software was used to quantitatively measure Dox release to the nucleus, thus confirming a statistically dramatically increase of the drug in the MCF-7/ADR nuclei after delivery by PPG-FA/siRNA/Dox with irradiation compared with free Dox, PPG-FA/Dox and PPG-FA/siRNA/Dox without irradiation groups (Fig. 5f).

### *In vitro* cytotoxicity studies

**Figure 6:**
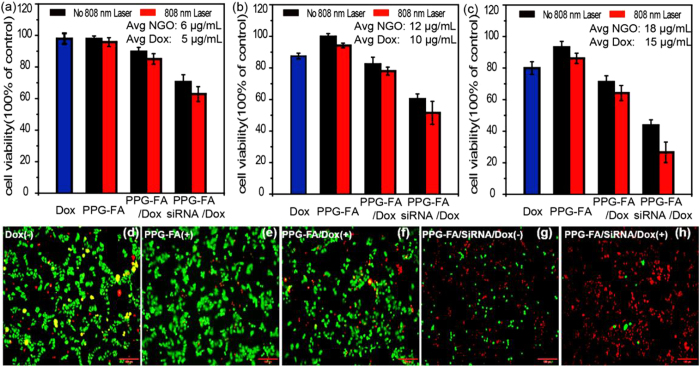
(**a–c**) Cell viability of the MCF-7/ADR cells after treatment for 24 h with Dox, PPG-FA, PPG-FA/Dox and PPG-FA/siRNA/Dox with or without 808 nm irradiation (2 W/cm2, 5 min) at various Dox concentrations (n=3). (**d–h**) Fluorescence images of Calcein AM/PI co-stained MCF-7/ADR cells (NGO equiv: 18 μg/mL, Dox equiv: 15 μg/mL). (+): 808nm irradiation (2 W/cm2, 5 min), (−): without irradiation. Scale bar 100 μm.

Finally, we investigated the synergistic effect of the co-delivery Dox and siRNA by PPG-FA to MCF-7/ADR cells under 808 nm irradiation. A Cell Counting Kit-8 (CCK-8) assay was performed to evaluate the cell viability of the MCF-7/ADR cells cultured with free Dox, PPG-FA, PPG-FA/Dox and PPG-FA/siRNA/Dox with or without 808 nm irradiation. Figure S3a shows the cytotoxicity of PPG-FA nanoparticles without irradiation at different NGO concentrations (6, 12, 18, 24, and 30 μg/mL). Significant cytotoxicity was not observed after incubation with PPG-FA at the NGO concentrations of 6, 12, and 18 μg/mL for 48 h. When the concentration increased to 24 and 30 μg/mL, slight cytotoxicity was observed due to the presence of the high molecular weight branched polyethylenimine, which implied that PPG-FA could be safely administered as a drug nanocarrier at low NGO concentrations. As shown Fig. 6a–c, the cell viability decreased with the increases in the NGO concentration for the PPG-FA group with irradiation, suggesting that minor cytotoxicity occurred. This finding may be explained by the photothermal effect of NGO. Respectively, higher cell cytotoxicity was observed for the PPG-FA/siRNA/Dox treatment compared with that observed for the free Dox and PPG-FA/Dox treatments at an equivalent Dox concentration with or without irradiation. As expected, all of the treatment groups showed dose-dependent toxicity, whereas the PPG-FA/siRNA/Dox system presented the greatest cytotoxicity up to a Dox concentration of 15 μg/mL, which corresponded with cell survival of less than 30% under irradiation. In summary, by the co-delivery of Dox and siRNA in the PPG-FA/siRNA/Dox system produced a strong synergistic photo-chemotherapy effect *in vitro*.

Their effective photo-chemotherapy effect was confirmed by co-staining with propidium iodide (PI) and Calcine AM assay (Fig. 6d–h and Fig. S3b–d). Red and green fluorescence from PI and Calcein AM corresponded to dead and live cells. As illustrated in Fig. S3b and c, there was no apparent cell death when the cells were treated with PBS (−) and PPG-FA (−) groups. Respectively, the dead cells gradually increased slightly for the Dox (−) (Fig. 6d), PPG-FA/Dox (−) (Fig. S3d) and PPG-FA/siRNA/Dox (−) (Fig. 6g) groups (with the equivalent of Dox concentration of 15 μg/mL), which is due to the increased cellular uptaken ability. It can also be observed that PPG-FA (+) and PPG-FA/Dox (+) group (Fig. 6e and f) caused same cells to die because of the photothermal effect of PPG-FA and the chemotherapeutic efficacy of Dox. Compared with the other groups, a large number of cells were destroyed and the dead cells obviously increased for the PPG-FA/siRNA/Dox (+) group due to the synergistic therapy, which was in a good agreement with CCK-8 assay.

**Figure 7:**
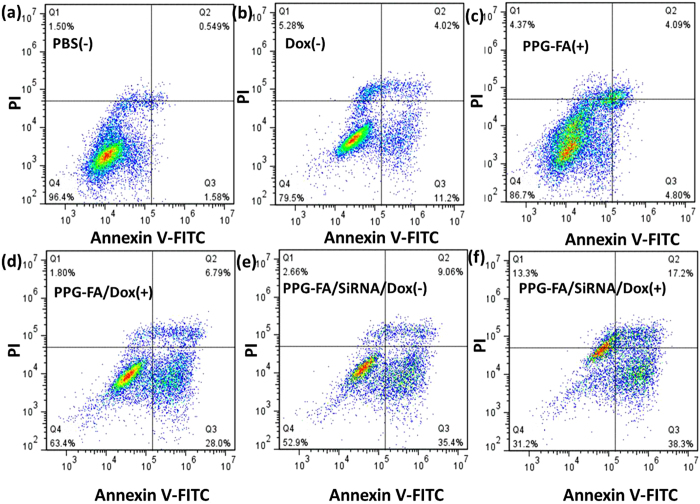
Apoptosis rate of MCF-7/ADR cells treated by PBS (−), Dox (−), PPG-FA (+), PPG-FA/Dox (+), PPG-FA/siRNA/Dox (−) and PPG-FA/siRNA/Dox (+) were tested by flow cytometry. They are measured by the average fluorescent intensity (FL) treated with Annexin V-FITC/PI. (+); 808nm irradiation (2 W/cm^2^, 5 min); (−); without irradiation.

Furthermore, fluorescein isothiocyanate (FITC)-conjugated Annexin V (Annexin V-FITC) apoptosis detection kit was performed by flow cytometry to evaluate the apoptotic rate. Propidium iodide (PI) was used as a nuclear stain for identifying late apoptotic and necrotic. MCF-7/ADR cells were treated with PBS (−), Dox (−), PPG-FA (+), PPG-FA/Dox (+), PPG-FA/siRNA/Dox (−/+) at equivalent of Dox concentration of 15 μg/ml. As revealed in Fig. 7, our results indicated that, after irradiation with light, PPG-FA/siRNA/Dox (+) (Fig. 7f) exhibited the highest total apoptotic ratio of 55.5% (a sum of the early apoptotic ratio of 38.3% and the late apoptotic ratio of 17.2%), which was significantly higher than other groups (2.1%, 15.2%, 8.9%, 34.8%, 44.4%, respectively, Fig. 7a–e). The results revealed PPG-FA/siRNA/Dox (+) group displayed effective photo-chemotherapy effect.

## Summary

In summary, we demonstrated that nanographene could be modified to serve as a dual carrier for delivering Dox and siRNA to overcome drug-resistant cancer cell. The PPG-FA carrier can load Dox *via* π–π stacking interactions and siRNA *via* electrostatic interactions. The nanocomplex exhibits high drug loading and siRNA affinity outside tumour cells, and displays a pH-responsive and heat-responsive Dox release behaviour, with faster Dox release rates observed under acidic pH condition. The Western blot analysis results demonstrated that the PPG-FA/siRNA system could mediate effectively gene silencing in MCF-7/ADR cells to defeat MDR and re-sensitize the cells to the chemotherapy drug Dox. Compared with free Dox or PPG-FA/Dox, PPG-FA/siRNA/Dox demonstrated significant cellular uptake, improved Dox penetration into the nuclei, and enhanced Dox cytotoxicity to the MCF-7/ADR cells. Significantly, under NIR light irradiation, the *in vitro* cell experiments indicated that the synergistic therapy was highly efficient for drug-resistant cancer therapy. Our results suggested that PPG-FA composite vesicles could be a powerful tool for drug delivery to achieve improved therapeutic efficacy and overcome drug resistance in combined photo-chemotherapy.

## Materials and Methods

### Materials

Graphite powder was purchased from Acros. KMnO_4_ (AR), NaNO_3_ (AR), linear chain PEG amine (5000), branched polyethylenimine 10000 (BPEI 10000), folic acid, EDC and NHS, were purchased from Sigma-Aldrich. Doxorubicin was purchased from Sangon Biotech (Shanghai, China). Modified RPMI-1640 Medium was purchased from HyClone. Fetal bovine serum (FBS), penicillin-streptomycin, and trypsin were obtained from Gibco. Propidium iodide, LysoTracker, MitoTracker and Hoechst were purchased from Molecular Probes (USA). The Cell Counting Kit-8 (CCK-8) and Calcein AM were purchased from Dojindo (Japan). The β-actin antibody was purchased from GeneTex (USA). The dialysis bags and ultrafiltration tubes (molecular weight cutoff: 10-kDa and 30-kDa) were purchased from Millipore. The P-gp siRNA, the scrambled siRNA and FAM-labelled siRNA were supplied by GenePharma (Shanghai, China).

### Synthesis of PPG, PPG-FA and PPG-FA/Dox

#### Preparation of PPG

PPG was prepared based on our previously reported methods^27^,^28^. Briefly, to strongly bind negatively charged siRNA *via* electrostatic interactions, we used the high molecular weight branched polyethylenimine (10000) instead of low molecular weight branched polyethylenimine (1800) to modify PG *via* using cross-linking reagents EDC and NHS.

#### Preparation of PPG-FA

Firstly, 40 mg EDC and 40 mg NHS were mixed in 4 mL FA (0.5 mg/mL, DMSO) solution and magnetically stirred at room temperature for 15 min, after activation, 4 ml PPG (1.0 mg/mL) solution was added in the solution to react at room temperature for 24 h. Finally, excess FA was removed by dialyzing against double distilled water (DD) for 24 h (molecular weight cutoff: 10-kDa). PPG-FA was obtained in an amount equal to NGO-PEG (0.5 mg/mL).

Preparation of PPG-FA/Dox. Dox was loaded onto PPG-FA *via* the following method. Briefly, 500 μL PPG-FA (0.5 mg/mL) and 500 μL Dox (1 mg/mL) were mixed in a 5 mL PBS solution and magnetically stirred at room temperature for 12 h. Then, excess Dox was removed by dialyzing the entire system against DD water until the filtrate was free of red colour.

### siRNA transfection

Preparation of PPG-FA/siRNA: The P-gp siRNA, scrambled siRNA and FAM-labelled siRNA were respectively dissolved in DEPC-treated water to achieve 0.1 mg/mL solution. The PPG-FA was blend with different siRNA solution at a weight ratio of 4:1 (NGO: siRNA), and incubated at room temperature for 30 min. It allows the electrostatic adsorption between negatively charged siRNA and positively charged PPG-FA. For preparation of PPG-FA/Dox/siRNA, the procedures were the same as that for PPG-FA/siRNA except using PPG-FA/Dox dispersed in DEPC-treated water.

### Materials characterization

The morphology and size of the PPG-FA/Dox were observed by AFM (BRUKER Dimension Icon) and TEM (JEM-1200EX 120KV). UV/vis spectra were collected on a P-360 nanophotometer. The absorbance at 490 nm was used as the characterization peak to confirm the successful conjugation of Dox and loading concentration. Fluorescence spectra were measured using NIR fluorescence spectrograph (Thermo Fisher) under 480 nm excitation.

### Photothermal conversion experiments

1 ml of PPG-FA solution is placed in a quartz cell and irradiated by 808 nm at a power density of 2 W/cm^2^ for 10 min, at pre-designed time intervals (2.5, 5.0, 7.5, 10.0 min), the temperature was measured by infrared thermometer.

### *In vitro* release study

The release behaviour of Dox was carried out at pH values of 7.4 and 5.0 over a time period of 48 h using the dialysis method. Briefly, 2 mL PPG-FA/Dox (80 μM) was placed in a dialysis bag with a molecular weight cutoff of 10-kDa. The dialysis bag was transferred in 20 mL of release medium at 37 °C and stirred for 48 h. At pre-designed time intervals (12, 24, 36 and 48 h), samples (0.4 mL) were periodically collected, and the amount of released Dox was analysed using P-360 nanophotometer UV/vis spectroscopy. For the photothermal release study, 2 mL PPG-FA/Dox was added to a 6-well plate, adjusted the pH of solution to 7.4 and 5.0, respectively. Then, at pre-designed time intervals (12, 24, 36 and 48 h), the samples was irradiated with 808 nm laser (2 W/cm^2^, 5 min). Next, the solution was placed in 30-kDa ultrafilter for removing the released Dox, the intercept solution was volumed to 2 mL and analysed using P-360 nanophotometer UV/vis spectroscopy.

### Cell culture

MCF-7 cell lines and MCF-7/ADR cell lines were cultured in RMPI-1640 with 10% FBS and 1% penicillin/streptomycin at 37 °C with 5% CO_2_.

### Internalization of PPG-FA

CLSM was performed to confirm the intracellular localization of PPG-FA. MCF-7/ADR cells (10 × 10^4^ cells/well) were cultured in 35-mm culture dishes with PPG-FA/Ce6 at 37 °C for 24 h. After washing three times with PBS, LysoTracker or MitoTracker was added to stain the lysosome or mitochondrion for 20 min, Hoechst was added to stain nucleus for 15 min. Finally, the cells were washed three times with PBS before capturing images by using CLSM (FLUOVIEWFV10i Olympus).

### Cellular uptake of siRNA-FAM and PPG-FA/siRNA-FAM

MCF-7/ADR cells (10 × 10^4^ cells/mL) were incubated with siRNA-FAM and PPG-FA/siRNA-FAM (siRNA equivalent of 2 μg) for 4 h, respectively. Then, each well was rinsed three times with PBS. Additionally, Hoechst was added to stain cell nuclei for 15 min, rinsed several times with PBS, and observed by CLSM. The signal intensity of the green channel, which reflects siRNA abundance, was calculated *via* Image J software (Image Lab 4.1, Bio-Rad, USA).

### Assessment of P-gp expression

The P-gp protein expression after drug treatment was evaluated by Western blot analysis. First, 20 μL of 0.1 μg/μL siRNA or scrambled siRNA was mixed with 500 μL PPG-FA (NGO equivalent of 8 μg), and then incubated 30 min at room temperature. MCF-7/ADR cells (2 × 10^5^ cells/well) were plated and exposed to the drugs (free siRNA, PPG-FA/siRNA and PPG-FA/scrambled siRNA) for 24 h. Then, the cells were washed three times with PBS, lysed by adding SDS sample buffer and scraped. Proteins were separated using SDS-PAGE gels and electrophoretically transferred to a polyvinylidene difluoride (PVDF) membrane. After blocking of the membranes in tris-buffered saline with 0.05% Tween-20 (TBST) for 1 h, these were overlaid with primary and secondary antibodies. Proteins were detected using chemiluminescence detection reagents, and an image was captured using Kodak imaging film. The relative gene expression was analyzed and normalized using β-actin as an endogenous control.

### Cellular uptake and intracellular concentration of Dox

Dox uptake in MCF-7/ADR cells was quantitatively evaluated *via* flow cytometry measurements. MCF-7/ADR cells (10 × 10^4^ cells/mL) were grown in 6-well plate and cultured with PBS, Dox, PPG-FA/Dox and PPG-FA/Dox/siRNA for 24 h (Dox equivalent of 5 μM). Then, the cells were washed three times with PBS, trypsinized, resuspended with medium, harvested using a centrifuge, and at last dispersed in 500 μL PBS. Flow cytometry analysis (BD FACS Verse) was performed to assess the fluorescence intensity of Dox.

CLSM was performed to investigate intracellular Dox distribution. MCF-7/ADR cells (10 × 10^4^ cells/mL) were exposed to free Dox (−), PPG-FA/Dox (−) and PPG-FA/siRNA/Dox (−/+) (Dox equivalent of 5 μM) on 35-mm glass-bottom dishes for 24 h. Then the cells were rinsed three times with PBS. Herein, the PPG-FA/siRNA/Dox (+) group was treated with 808 nm irradiation (2 W/cm^2^, 5 min) at 20 h. Subsequently, cell nuclei were stained with Hoechst for 15 min, rinsed several times with PBS, and observed *via* CLSM (FLUOVIEWFV10i Olympus). We also used Image J software to analyze the nuclear fluorescence of Dox.

### *In Vitro* Treatment

100 μL of cell suspension (5 × 10^3^ cells/well) was seeded in a 96-well plate and incubated for 24 h. Subsequently, the cells were exposed to various concentrations of Dox, PPG-FA, PPG-FA/Dox and PPG-FA/siRNA/Dox. After incubation for 24 h, the cells were treated with two ways. One sample group was treated with 10 μL CCK-8 solution and incubated for 2 h, and the absorbance at 450 nm was recorded using a Multiskan Spectrum (Thermo Fisher). The other sample group was treated with 808 nm irradiation (2 W/cm^2^, 5 min) followed by viability measurements at 24 h. The following formula was applied to calculate the cell viability: cell viability (%) = (mean absorbance value of treatment group/mean absorbance value of control) * 100%.

The synchronous effect of PPG-FA/siRNA/Dox for MCF-7/ADR cells was further verified using Calcein AM/PI. Cells (20 × 10^4^ cells/well) were seeded in 6-well plates and incubated overnight. Then, treated by PBS (−), Dox (−), PPG-FA (−/+), PPG-FA/Dox (−/+), and PPG-FA/siRNA/Dox (−/+) at an equivalent dosage (NGO 18 μg/mL; Dox 15 μM ) for 24 h, then cells were irradiated with 808 nm irradiation (2 W/cm^2^, 5 min) or no laser. Subsequently, the cells were incubated with Calcein AM (2 μM) and PI (1 μM) for 30 min and observed by fluorescence microscop (DMLRB inverted microscope, Leica).

### Apoptosis assay

MCF-7/ADR cells (20 × 10^4^ cells/well) were incubated with PBS (−), Dox (−), PPG-FA (+), PPG-FA/Dox (+) and PPG-FA/siRNA/Dox (−/+) at an equivalent dosage (NGO 18 μg/mL; Dox 15 μM) for 36 h in 6-well plates. Then, the cells were washed three times with PBS, re-suspended in 100 μL of binding buffer and stained with 2 μL of Annexin V-FITC and 2 μL of PI for 15 min at room temperature in the dark. After being stained, the cells were detected by flow cytometry to make clear the cell death mode.

## Additional Information

**How to cite this article**: Zeng, Y. *et al*. Multifunctional Nanographene Oxide for Targeted Gene-Mediated Thermochemotherapy of Drug-resistant Tumour. *Sci. Rep.* **7**, 43506; doi: 10.1038/srep43506 (2017).

**Publisher's note:** Springer Nature remains neutral with regard to jurisdictional claims in published maps and institutional affiliations.

## Supplementary Material

Supplementary Information
