# Supplementary material for: Multifunctional Nanographene Oxide for Targeted Gene-Mediated Thermochemotherapy of Drug-resistant Tumour
Source: Sci Rep. 2017 Mar 8;7:43506. doi: 10.1038/srep43506 (PMC5341118; doi:10.1038/srep43506)
Supplement: Supplementary Information [file srep43506-s1.pdf]

Supporting Information

Multifunctional Nanographene Oxide for Targeted Gene-Mediated Thermochemotherapy of Drug-resistant Tumour

Yiping Zeng [1], *Zhangyou Yang* [1,*], *Hong Li* [1], *Yuhui Hao* [1], *Cong Liu* [1], *Lin Zhu* [1], *Jing Liu* [1], *Binghui Lu* [1] *and Rong Li* [1,*]

[1]State Key Laboratory of Trauma Burns and Combined Injury, Institute of Combined Injury, Chongqing Engineering Research Center for Nanomedicine, College of Preventive Medicine, Third Military Medical University, Chongqing 400038, China.

* Address correspondence to oyzhangyou@126.com, lrong361@126.com.

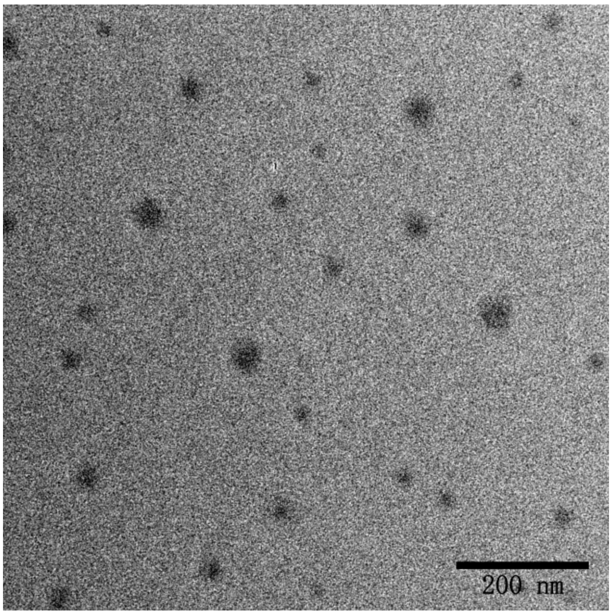

Figure S1 HR-TEM image of PPG-FA/Dox.

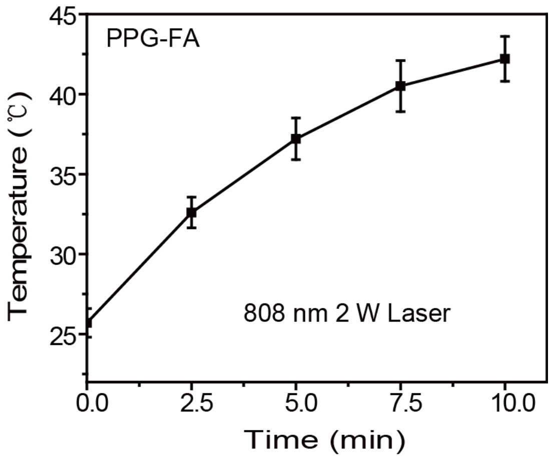

Figure S2 photothermal capacity of PPG-FA upon irradiation with 808 nm laser at a power density of 2 W/cm^2. Data are presented as the mean values ± S.D (n = 3).

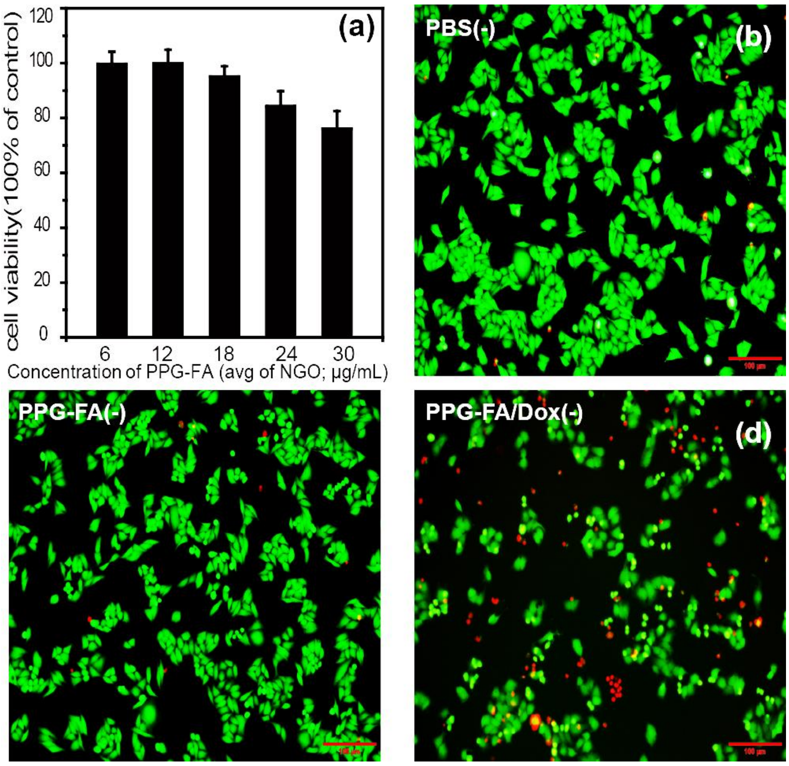

Figure S3 (a) Cell viability values of MCF-7/ADR cells incubated with PPG-FA without 808 nm irradiation at different concentration of PPG-FA. (b-d) Fluorescence images of CalceinAM-PI costained MCF-7/ADR cells incubated with PBS (-) (b), PPG-FA (-) (c) and PPG-FA/Dox (-) (d) (NGO equiv: 18 μg/ml, Dox equiv: 15 μg/ml). The meaning of (-) is the treatment without 808nm irradiation. Magnification 100 ×; scale bar 100 μm.
